# Dose-response relationship between Life's Essential 8 score and COPD risk: the NHANES cohort study 2007–2018

**DOI:** 10.3389/fmed.2025.1446782

**Published:** 2025-01-23

**Authors:** Qian Huang, Quan Yuan, Wenqiang Li, Xiaoyu He, Qian He, Zhiping Deng

**Affiliations:** ^1^Dazhou Dachuan District People's Hospital (Dazhou Third People's Hospital), Dazhou, Sichuan, China; ^2^Zigong First People's Hospital, Zigong, Sichuan, China; ^3^Department of Clinical Medicine, North Sichuan Medical College, Nanchong, Sichuan, China; ^4^Department of Gynecology and Obstetrics, West China Second University Hospital, Key Laboratory of Birth Defects and Related Diseases of Women and Children (Sichuan University) of Ministry of Education, Sichuan University, Chengdu, Sichuan, China

**Keywords:** CVH, COPD, dose-response relationship, LE8 score, health behavioral factors, health factors

## Abstract

**Objective:**

This study aims to discuss the dose-response relationship between the Life's Essential 8 (LE8) score and chronic obstructive pulmonary disease (COPD).

**Methods:**

We screened data from the National Health and Nutrition Examination Survey (NHANES) database for the years 2007–2018. Logistics regression analysis and subgroup analysis were used to explore the relationship between cardiovascular health (CVH) and COPD based on the LE8 score. Additionally, restricted cubic spline (RCS) plots were drawn to visually display the dose-response relationship.

**Results:**

A total of 12,517 participants were included, of which 835 had COPD. After multivariable adjustment, the LE8 score was found to be linearly and inversely associated with the risk of developing COPD. A similar relationship was observed in the scores for health behavior factors, whereas the relationship was weaker for health factors. The RCS plots visually demonstrated the aforementioned dose-response relationship. Moreover, subgroup analyses showed that this relationship remained robust across different groups.

**Conclusion:**

LE8 scores are inversely and linearly associated with the risk of developing COPD. Higher LE8 scores can reduce the risk of developing COPD in individuals over 40 years old, especially concerning health behavior factors.

## 1 Introduction

Cardiovascular] health (CVH) is particularly important for human lifespan and quality of life. Cardiovascular disease (CVD) is a prevalent and high-mortality chronic disease worldwide, affecting over 500 million people globally, with approximately 20 million deaths attributed to it each year (1). In 2010, the American Heart Association (AHA) first introduced Life's Simple 7 as the criteria for describing CVH, which includes physical activity, smoking, diet, body mass index, blood glucose, blood lipids, and blood pressure (2). In 2022, sleep health was added to Life's Simple 7 score, which was renamed Life's Essential 8 (LE8) score (3). Up to now, LE8 score, which has been proven to be associated with the incidence and mortality of various diseases (4–7). Therefore, it is evident that the LE8 score can better assess CVH status, which is advantageous for analyzing the dose-response relationship between CVH and diseases.

Chronic obstructive pulmonary disease (COPD) is a common chronic respiratory disease characterized by persistent and irreversible airflow limitation (8). Currently, according to statistics, it has become the fourth leading cause of death globally, with projections indicating that by 2030 it will rise to the third leading cause of death (9, 10). Most people are diagnosed over the age of 40 years (11). COPD and CVD often coexist, with their pathophysiology mutually influencing each other (12). CVD causes dyspnoea (heart failure leading to pulmonary oedema) and reduces exercise capacity (13, 14). Therefore, it may lead to the development or acute exacerbation of COPD. Previous study has shown that patients with ischemic heart disease (IHD) combined with COPD have poorer exercise capacity and are more prone to acute exacerbations of respiratory difficulties compared to those with COPD alone. Additionally, the combination of IHD and COPD increases the risk of patients mortality (15). Therefore, we can see that there exists a complex interaction between CVD and COPD.

In conclusion, there exists a certain degree of correlation between CVH and COPD. But so far, no studies have explored the dose-response relationship between CVH and the risk of developing COPD. Therefore, based on the National Health and Nutrition Examination Survey (NHANES) database, utilizing the LE8 score, we investigated the dose-response relationship between CVH and COPD. Our research findings can provide reference for clinical practice and individual lifestyle behaviors, with the aim of improving people's quality of life and health.

## 2 Research design and methods

### 2.1 Study population

FNHANES database (https://wwwn.cdc.gov/nchs/nhanes/Default.aspx) through complex sampling design and weighting methods to obtain a nationally representative sample. NHANES operates on a 2-year cycle, surveying approximately 5,000 individuals nationally each year. Therefore, it has advantages such as rich information sources, extensive tracking data, diverse content, and authentic data. All participants signed informed consent forms provided by the NHANES Ethics Review Committee.

**Figure 1:**
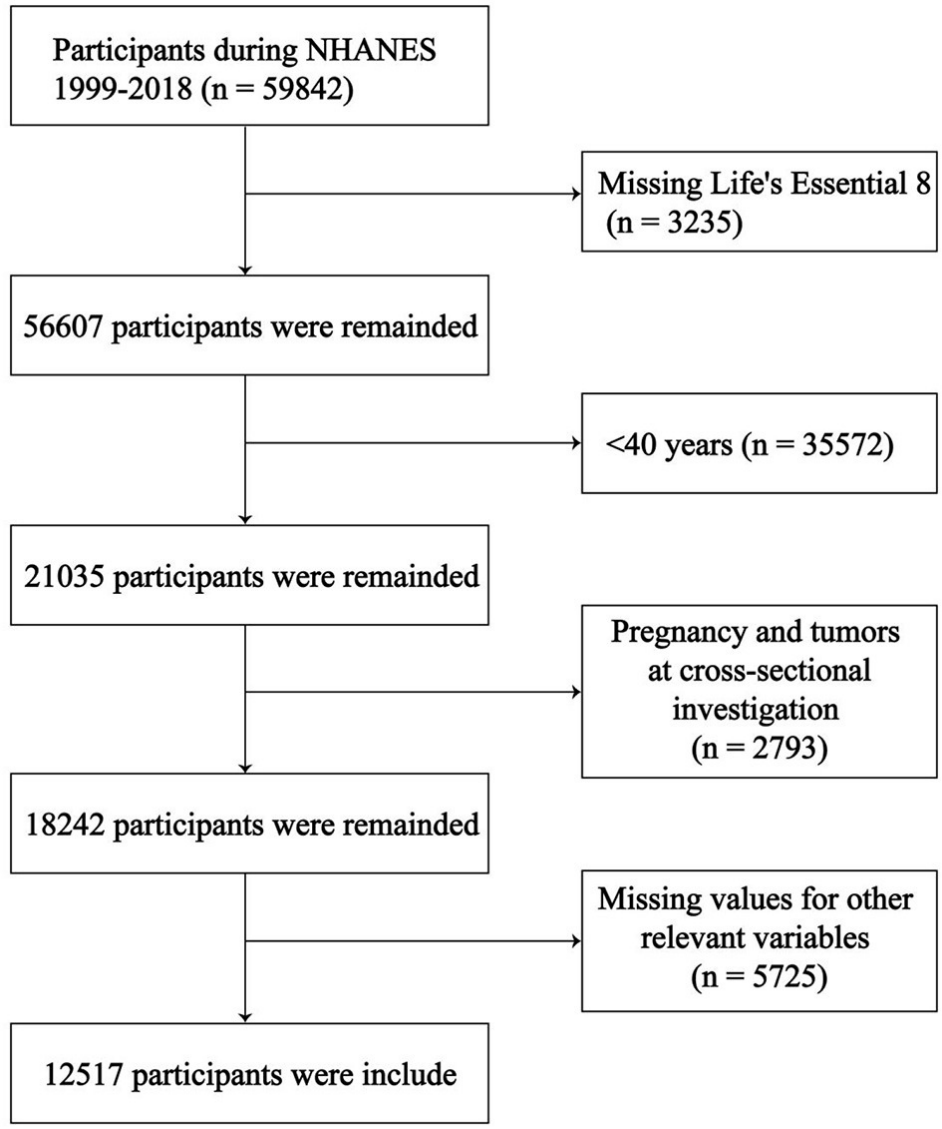
Study population screening process.

We selected the 2007–2018 NHANES database population (*n* = 59,842). The following relevant data were excluded: (1) Missing LE8 score (*n* = 3,235); (2) < 40 years old (*n* = 35,572); (3) Pregnant or with a tumor at the time of the cross-sectional survey (*n* = 2,793); (4) Missing values for other relevant variables (*n* = 5,725). Ultimately, we included 12,517 participants for analysis, 835 of whom had COPD (Figure 1).

### 2.2 Definition of COPD

COPD was judged based on pulmonary function tests, COPD questionnaire reports (MCQ160G, MCQ160P) and medication use. COPD was judged to be included in the study if one of the following three items were met. As follows:

1. FEV1/FVC < 0.7 after inhaled bronchodilators.
2. Have you been diagnosed with emphysema in the past.
3. Are COPD medications used (leukotriene regulators, inhaled corticosteroids, selective phosphodiesterase-4 inhibitors, mast cell stabilizers)? In addition, they have a history of smoking or chronic bronchitis and are over 40 years old.

### 2.3 Description of CVH

In this study, CVH was described using a LE8 score according to the AHA definition (3). The LE8 score is composed of health behavior factors (physical activity, smoking, diet, and sleep disorders) and health factors (blood sugar, lipids, blood pressure and body mass index). Each of these 8 indicators is assigned a score ranging from 0 to 100. The LE8 score is the average of the scores obtained for each item added together. Detailed algorithms for calculating the LE8 scores for each of the metrics to NHANES data have been previously published (3, 16, 17). Based on the total score, the LE8 score is categorized into high level (80–100), moderate level (50–79), and low level (0–49) (3).

### 2.4 Research variables

Demographic variables included age (40–64 or ≥65 years), sex (Male or Female), race (White or Other), and marriage (Married/Living with Partner, Widowed/Divorced/Separated or Never married). Socioeconomic variables included education level (Lower than high school, High school diploma or More than high school), Poverty Income Ratio (PIR) (< 1.3, 1.3–3.5 or >3.5), and family insurance (With or Without). PIR ranged from 0 (no family income) to 5 (family income at least five times the annual federal poverty level). Environmental exposure variables included blood lead ([0.05, 0.9], [0.9, 1.34], [1.34, 2.04], [2.04, 38.9]) and blood mercury ([0.11, 0.47], [0.47, 0.88], [0.88, 1.77], [1.77, 85.7]). In addition, asthma (No or Yes) is included.

### 2.5 Statistical analysis

The NHANES database used a complex sampling design and constructed sample weights to obtain a nationally representative sample. Appropriate weights were selected based on the research factors and outcomes of this study, and the following statistical analyses were based on the weighting of the data. All statistical analyses were performed using R software version 4.2.1, and a two-tailed *P* < 0.05 was considered statistically significant. The variables of our study were converted into categorical variables, expressed as percentages, and compared between groups using chi-square test. Subsequently, we analyzed the relationship between LE8 scores and COPD by using propensity score matching (PSM) to match the three levels of LE8 scores two by two so that the differences between groups were as consistent as possible. In order to try to satisfy the matching principles of PSM (maximum use of sample size and consistency of covariates between groups), we found that LE8 scores were best analyzed after several attempts to match low and moderate LE8 scores in a 1:1 ratio, and moderate and high LE8 scores in a 2:1 ratio. Simultaneously, logistic regression analysis was used to assess the odds ratio (OR) of LE8 scores to COPD in terms of categorical variables and 95% confidence intervals (CI) were calculated.

During the analysis process, we constructed three models. Model 1 does not adjust any factors. Model 2 adjusts for sex, age, race, education, and marriage. Model 3 adjusts the insurance and PIR based on Model 2. Restricted cubic spline (RCS) is a commonly used method for studying the non-linear relationship between continuous variables and outcome variables, primarily designed to reflect the trend between the two (*P* for non-linear < 0.05 indicates a non-linear correlation; *P* for non-linear > 0.05 suggests no non-linear correlation, meaning a linear correlation in a sense). We further developed a RCS to investigate the potential dose-response relationship between LE8 scores and COPD. Conduct subgroup analysis by sex, age, race, PIR, BMI, and education level to assess whether there were any interaction effects on the aforementioned relationship. Additionally, sensitivity analyses were performed by incorporating factors such as blood lead, blood mercury, and asthma to assess the robustness of the relationship between LE8 score and COPD.

## 3 Results

### 3.1 Baseline characteristics

A total of 12,517 participants were included in the analysis, 835 of whom had COPD. From Table 1, we can see that COPD is more likely to occur in people aged 45–64, with high education, married/living with partner and family insurance, and there is little difference in incidence between genders. LE8 score, an international measure of CVH, is more likely to develop COPD at moderate levels (50–79) and lowest at high levels (80–100).

**Table 1:** Baseline characteristics of the study population.

**Variable**	**Total**	**Non-COPD**	**COPD**	***P***
Age group (%)				< 0.001
40–64	8,726 (75.83)	8,246 (76.55)	480 (65.05)	
≥65	3,791 (24.17)	3,436 (23.45)	355 (34.95)	
Sex (%)				0.04
Female	6,505 (53.13)	6,136 (53.58)	369 (46.37)	
Male	6,012 (46.87)	5,546 (46.42)	466 (53.63)	
Race (%)				< 0.001
Other	6,954 (27.47)	6,643 (28.08)	311 (18.28)	
White	5,563 (72.53)	5,039 (71.92)	524 (81.72)	
Marital status (%)				0.02
Married/Living with Partner	7,896 (68.31)	7,421 (68.54)	475 (64.83)	
Never married	1,069 (7.64)	1,007 (7.75)	62 (6.00)	
Widowed/Divorced/Separated	3,552 (24.05)	3,254 (23.71)	298 (29.17)	
Education (%)				< 0.001
High school diploma	2,931 (24.51)	2,713 (24.33)	218 (27.14)	
Lower than high school	3,019 (14.80)	2,757 (14.27)	262 (22.69)	
More than high school	6,567 (60.70)	6,212 (61.41)	355 (50.17)	
Poverty income ratio (%)				< 0.001
< 1.3	3,580 (17.89)	3,254 (17.25)	326 (27.48)	
1.3–3.5	4,746 (34.04)	4,434 (33.79)	312 (37.80)	
>3.5	4,191 (48.06)	3,994 (48.96)	197 (34.72)	
Insurance (%)				0.14
No	2,028 (12.62)	1,940 (12.78)	88 (10.19)	
Yes	10,489 (87.38)	9,742 (87.22)	747 (89.81)	
Life's Essential 8 (LE8) score (%)				< 0.001
Low (0–49)	2,110 (13.58)	1,845 (12.75)	265 (25.99)	
Moderate (50–79)	8,629 (68.12)	8096 (68.10)	533 (68.48)	
High (80–100)	1,778 (18.30)	1741 (19.16)	37 (5.53)	

### 3.2 PSM and analyzing results

To ensure that baseline characteristics were as consistent as possible between groups, we applied PSM to match each pair of LE8 score levels. Before matching, apart from sex and age, there were significant differences in marriage, race, PIR, education level, and household insurance between the low and moderate LE8 score groups (*P* < 0.05). However, after 1:1 PSM, there were 2,098 participants in each of the low and moderate LE8 score groups, and there were no statistically significant differences in baseline characteristics between the two groups (*P* > 0.05) (see Supplementary Table 4). Logistic regression analysis was then used to compare the risk of COPD occurrence between the low and moderate LE8 score groups. The results indicated that the risk for COPD in the moderate LE8 score group was 61% lower than in the low LE8 score group [OR (95% CI) 0.39 (0.29, 0.53], *P* < 0.0001) (see Supplementary Table 6). Between moderate and high levels, the baseline difference before matching was large (*P* < 0.05), and after multiple PSM attempts, we found that the best results were obtained with a 2:1 matching ratio, with 3,580 and 1,790 participants in each of the moderate and high level groups, with no statistical difference in the distribution of the remaining factors between the two groups (*P* > 0.05), with the exception of marriage (see Supplementary Table 5). Differences in the risk of COPD occurrence between LE8 scores in the moderate and high level groups were also compared by logistic regression analysis. The results showed that the risk of developing COPD was 58% lower at high levels than at moderate levels [OR (95% CI) 0.42 (0.26, 0.67), *P* < 0.001] (see Supplementary Table 6). Thus, our post-PSM analysis showed that the risk of COPD decreased with increasing levels of LE8 score.

### 3.3 Association of LE8 score with COPD

**Figure 2:**
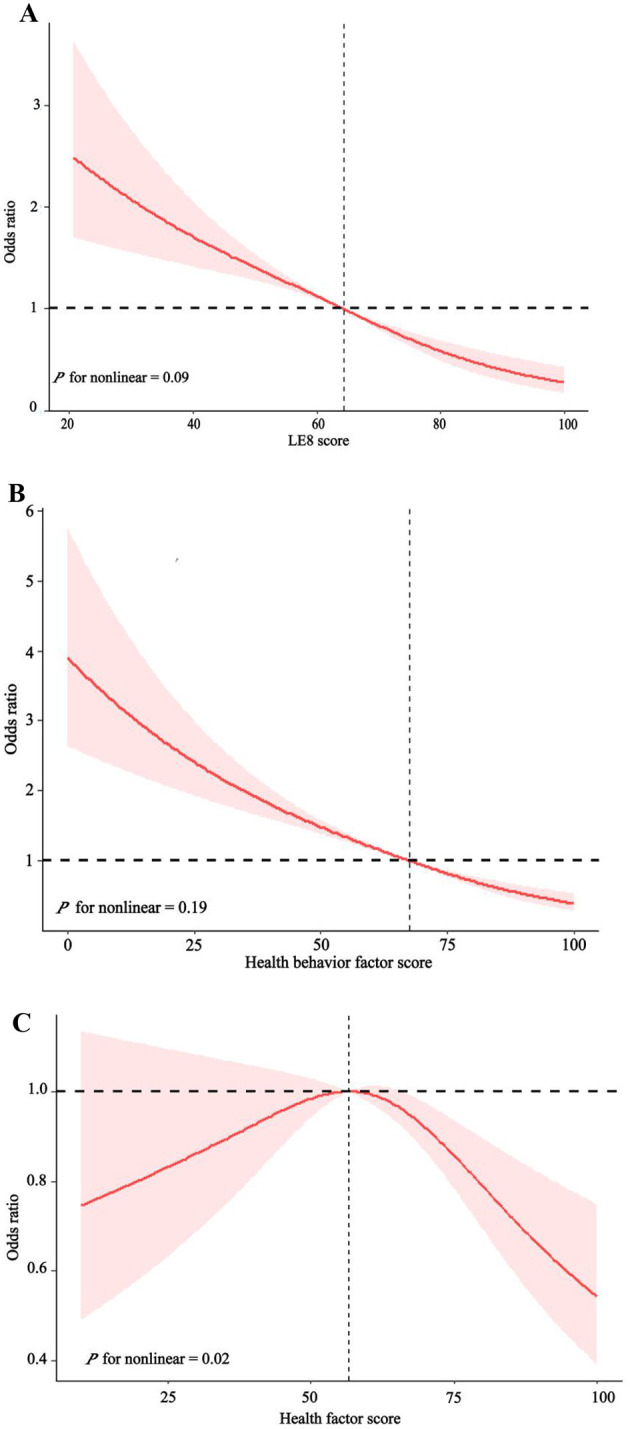
The dose-response relationship between LE8 score **(A)**, health behavior factors score **(B)**, health factors score **(C)**, and Chronic obstructive pulmonary disease (COPD).

As can be seen from Table 2, we divided LE8 score into three groups [high level (80–100), moderate level (50–79), and low level (0–49)] to explore its potential response relationship with COPD. We found that the risk of COPD decreased as LE8 score levels increased, regardless of sex, age, race, or socioeconomic status. In the complete model, those with moderate LE8 score had a 45% lower risk of COPD compared to those with low LE8 score [OR 0.55, 95%CI (0.45, 0.67), *P* < 0.001]. Those with high levels of LE8 score had an 82% reduction in COPD risk [OR 0.18, 95%CI (0.11, 0.31), *P* < 0.001], and high levels had about 37% more reduction than moderate levels. Figure 2A showed a linear relationship between LE8 score and the risk of COPD occurrence (P for non-linear = 0.09), with a gradual decrease in the risk of COPD occurrence as LE8 score increased. The above results indicate that CVH is closely associated with COPD, emphasizing that maintaining a high level of LE8 score is beneficial in reducing the risk of developing COPD.

**Table 2:** Association between the LE8 score and Chronic obstructive pulmonary disease (COPD).

	**Model 1 OR (95%CI)**	***P-*value**	**Model 2 OR (95%CI)**	***P-*value**	**Model 3 OR (95%CI)**	***P-*value**
**LE8 score**						
Low (0–49)	reference	/	reference	/	reference	/
Moderate (50–79)	0.49 (0.41, 0.60)	< 0.001	0.51 (0.41, 0.62)	< 0.001	0.55 (0.45, 0.67)	< 0.001
High (80–100)	0.14 (0.09, 0.23)	< 0.001	0.16 (0.10, 0.27)	< 0.001	0.18 (0.11, 0.31)	< 0.001
*P* for trend	< 0.001	/	< 0.001	/	< 0.001	/
**Health behaviors score**						
Low (0–49)	reference	/	reference	/	reference	/
Moderate (50–79)	0.53 (0.42,0.67)	< 0.001	0.55 (0.43,0.70)	< 0.001	0.58 (0.46,0.74)	< 0.001
High (80–100)	0.25 (0.19,0.33)	< 0.001	0.27 (0.19,0.37)	< 0.001	0.29 (0.21,0.40)	< 0.001
*P* for trend	< 0.001	/	< 0.001	/	< 0.001	/
**Health factors score**						
Low (0-49)	reference	/	reference	/	reference	/
Moderate (50-79)	0.86 (0.66,1.11)	0.25	0.89 (0.68,1.15)	0.37	0.94 (0.72,1.24)	0.67
High (80-100)	0.54 (0.38,0.76)	< 0.001	0.62 (0.44,0.88)	0.01	0.67 (0.47,0.94)	0.02
*P* for trend	< 0.001	/	0.008	/	0.026	/

### 3.4 Association of health behavior factors with COPD

As can be seen in Table 2, all models showed a decreasing trend in health behavior factor scores and COPD (*P* < 0.001). In the full model, those with high levels of health behavior factor scores had a 71% [OR 0.29, 95%CI (0.21, 0.40), *P* < 0.001] lower risk of developing COPD compared to those with low levels of health behavior factor scores. Figure 2B shows a linearly decreasing relationship between the health behavior factor score and the risk of COPD (P for non-linear = 0.19), emphasizing that a high level of health behavior score in LE8 score is conducive to reducing the risk of COPD.

### 3.5 Association of health factors with COPD

As can be seen from Table 2, all models show that the health behavior factor in LE8 score is closely related to COPD. There was no significant difference in the impact of low and moderate health factor scores on COPD, while high health factor scores significantly reduced COPD risk than low health factor scores. In the complete model, those with high levels of health factors had a 33% [OR 0.67, 95%CI (0.47, 0.94), *P* = 0.02] lower risk of COPD compared to those with low levels of health factors. Figure 2C shows that the health factor score is negatively correlated with the risk of COPD (P for non-linear = 0.004).

### 3.6 Subgroup analysis and sensitivity analyses

**Figure 3:**
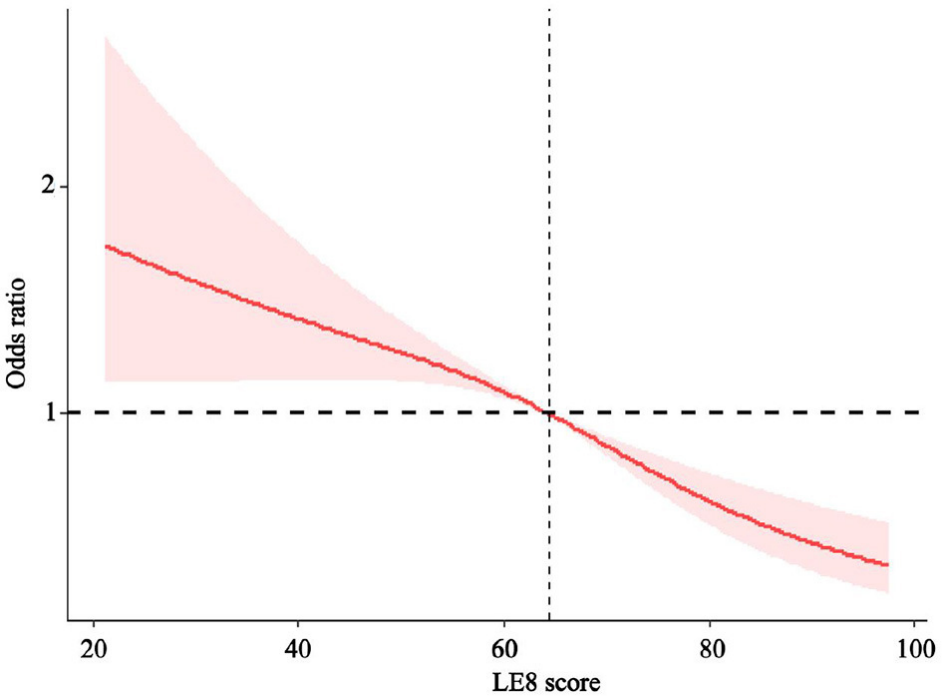
Sensitivity analysis of the dose-response relationship between LE8 score.

Subgroup analysis showed this decreasing relationship to be robust and unaffected by gender, age, race, PIR, household insurance, and education level (Table 3, P for interaction >0.05). However, this relationship may not exist at the high school diploma or in the uninsured group. Then, we conducted a sensitivity analysis by including blood lead, blood mercury, and asthma. After excluding participants with missing data, a total of 10,437 participants were included in the final analysis, with baseline characteristics detailed in Supplementary Table 7. The final model from the multivariate logistic regression analysis still demonstrated a negative correlation between LE8 score and the risk of COPD occurrence (P for trend < 0.0001, in Supplementary Table 8). The RCS showed a non-linear decreasing relationship (non-linear *P* = 0.0417), which is not entirely consistent with previous results, but the overall trend is similar (Figure 3).

**Table 3:** Subgroup analysis of the association between the LE8 score and Chronic obstructive pulmonary disease (COPD).

**Character**	**0–49**	**50–79**	***P***	**80–100**	***P***	***P* for interaction**
Sex						0.07
Female	ref	0.46 (0.35,0.62)	< 0.001	0.19 (0.11,0.35)	< 0.001	
Male	ref	0.51 (0.38,0.68)	< 0.001	0.08 (0.04,0.17)	< 0.001	
Age group						0.11
40–64	ref	0.48 (0.36,0.64)	< 0.001	0.11 (0.05,0.21)	< 0.001	
≥65	ref	0.53 (0.38,0.73)	< 0.001	0.29 (0.15,0.59)	< 0.001	
Race						0.91
other	ref	0.49 (0.35,0.68)	< 0.001	0.11 (0.05,0.27)	< 0.001	
white	ref	0.47 (0.38,0.60)	< 0.001	0.13 (0.08,0.23)	< 0.001	
Education						0.05
High school diploma	ref	0.78 (0.50,1.22)	0.27	0.53 (0.18,1.60)	0.26	
Lower than high school	ref	0.45 (0.31,0.67)	< 0.001	0.11 (0.03,0.38)	< 0.001	
More than high school	ref	0.43 (0.32,0.59)	< 0.001	0.11 (0.06,0.20)	< 0.001	
Poverty income ratio						0.31
< 1.3	ref	0.56 (0.37,0.83)	0.01	0.27 (0.11,0.63)	0.003	
1.3–3.5	ref	0.43 (0.32,0.58)	< 0.001	0.14 (0.03,0.70)	0.02	
>3.5	ref	0.75 (0.47,1.21)	0.24	0.17 (0.09,0.32)	< 0.001	
Insurance						0.6
No	ref	0.68 (0.37,1.25)	0.21	0.26 (0.03,2.37)	0.23	
Yes	ref	0.47 (0.38,0.58)	< 0.001	0.13 (0.08,0.21)	< 0.001	

## 4 Discussion

We utilized the NHANES database to conduct a cross-sectional survey investigating the association and potential dose-response relationship between LE8 score and COPD among middle-aged and elderly individuals. In this demographic, regardless of age, gender, race, marital status, or socioeconomic status, there is a linear inverse relationship between LE8 scores and COPD. The LE8 consists of health behavior factors and health factors. A similar relationship exists between health behavior scores and COPD. However, the relationship weakens for health factors but remains significant at higher percentiles.

The LE8 score can assess the status of CVH and is an ideal metric for quantifying CVH. To the best of our knowledge, previous studies have shown that CVH described by LE8 score is associated with a variety of diseases (non-alcoholic fatty liver disease, chronic kidney disease, depression, and dementia) (7, 16, 18, 19). However, there are no existing studies on the dose-response relationship between LE8 scores and COPD. And CVD and COPD occurs more often after middle age. Therefore, we explored the relationship between LE8 score and COPD in middle-aged and elderly people for the first time in conjunction with the NHANES database, going to fill this gap, which can help guide the clinic to prevent the occurrence of COPD at an early stage, and reduce the burden of healthcare for the society.

Nowadays, the mechanism between LE8 score and COPD is not clear. However, studies have shown that blood glucose, blood lipids, body mass index, sleep and other metabolic indicators and lifestyle are related to COPD or decreased lung function (20–25). It has been shown that diabetic patients have poorer baseline lung function than the general population and that for every 1 mmol/L increase in fasting glucose, FVC, FEV1, FVC%, and FEV1% (indicators of lung function) decrease by 25 ml, 13 ml, 0.71%−1.03%, and 0.46%−0.72%, respectively (24). Hyperglycaemia induces mitochondrial damage leading to oxidative stress generating excess reactive oxygen species (ROS) causing lung injury (26). Reduced levels of Nuclearfactor erythroidderived 2-like 2 (Nrf2) in diabetic patients lead to a reduction in endogenous antioxidant factors causing systemic oxidative stress (27). Smoking is the most important environmental pathogen for COPD. Smoking not only causes chronic inflammation that directly contributes to COPD, but also reduces insulin action causing hyperglycaemia in the body which indirectly leads to decreased lung function (28). Body weight is strongly associated with COPD. A large prospective study from China showed that low body mass index or abdominal obesity increased the risk of COPD (25). Patients with abdominal obesity have a high amount of visceral fat, which is a pro-inflammatory mediator that attracts inflammatory cells and amplifies the inflammatory process leading to alterations in the structure of the small airways (29). Furthermore, underweight individuals may have a predisposition to respiratory infections and muscle loss to develop COPD (30). The LE8 score is a composite indicator that includes all of these factors, and thus is strongly associated with the presence of COPD. Additionally, the LE8 score is closely related to CVH. Multiple studies have indicated that CVD may physiologically influence the occurrence of COPD (13, 14, 31, 32). CVD could lead to reduced pulmonary vascular perfusion and hyperventilation, subsequently lowering gas exchange efficiency and contributing to the development of COPD (13, 14, 31, 32).

Studies have shown that several drugs are potential drugs for the treatment of COPD (33–37). Xue et al. reviewed a number of studies from 2008–2019 and found that statins can increase exercise capacity and improve lung function in COPD patients (33). Francesca et al. constructed a mouse model and found that metformin prevented cigarette smoke-induced lung inflammation delaying the progression of emphysema (34). Prolonged low- or moderate-intensity physical activity is beneficial in COPD, increasing aerobic capacity and improving resistance to fatigue (37). However, most of the previous studies have focused on the effect of individual treatment modalities on COPD and lacked all-round lifestyle recommendations. The LE8 score is a comprehensive, simple, and easily accessible indicator that can be better applied to clinical practice. Our study found that LE8 score, as an ideal indicator of CVH, was linearly and negatively correlated with COPD, and that maintaining a high level of LE8 score could reduce the risk of COPD. LE8 score is helpful for clinical assessment of the risk of COPD, which is conducive to the development of effective lifestyle behaviors to reduce the incidence of COPD and thus reduce the socio-economic burden. Based on the detailed scoring criteria of the LE8 score, it is evident that middle-aged and elderly individuals can significantly reduce their risk of COPD by adopting: (1) A Mediterranean diet pattern. (2) Maintaining sleep duration between 7 and < 9 h. (3) Engaging in at least 150 min of moderate-intensity exercise per week. (4). Keeping their body mass index (BMI) below 25. (5) Reducing or avoiding smoking. (6) Ensuring that non-high-density lipoprotein (HDL) cholesterol levels are below 3.4 mmol/L. (7) Maintaining blood pressure at or below 120/80 mmHg. (8) Keeping fasting blood glucose < 5.6 mmol/L (or HbA1c < 5.7%) for those without a history of diabetes. (9) Keeping HbA1c < 7% for those with a history of diabetes. To facilitate these health practices, we recommend that middle-aged and elderly individuals schedule annual comprehensive health check-ups, including pulmonary function tests, blood pressure, blood glucose, and lipid profile measurements. Early detection and management of potential health issues can help formulate personalized, actionable health advice for preventing COPD.

This study includes a large nationally representative sample and subgroup analysis, which has relatively stable results and can be generalized to a wider population. For the first time, the relationship between LE8 score and COPD was assessed, and it was found that maintaining a high level of LE8 score reduced the risk of developing COPD, identifying a potential dose-response relationship between LE8 score and COPD, with similar associations for health behavioral factor scores. Inevitably, there are some limitations to this study. First, health behavior factors were presented as self-reports, which may be subject to recall bias and self-report bias. Second, our study was a cross-sectional investigative study and therefore cannot demonstrate causality and temporality between LE8 score and COPD. Third, we studied people over 40 years of age, so the findings may not be applicable to people under 40 years of age. While the exclusion of people under the age of 40 may improve the focus and statistical validity of the study, it may also lead to limitations in the applicability and generalizability of the findings to younger populations. Future studies may need to balance these issues in their design to ensure that the findings serve the public health needs of different age groups more comprehensively.

## 5 Conclusion

The LE8 scores shows a linear inverse relationship with the risk of developing COPD, with health behavior factors demonstrating a similar relationship. However, this relationship is weaker for health factors. Maintaining high LE8 scores can reduce the risk of developing COPD. So LE8 score has the potential to become a simple assessment tool for evaluating the risk of COPD, but further research is needed to explore the longitudinal and causal relationships between LE8 scores and COPD.

## Data Availability

The datasets presented in this study can be found in online repositories. The names of the repository/repositories and accession number (s) can be found below: the dataset for this study can be found on the NHANES website NHANES - National Health and Nutrition Examination Survey Homepage (cdc.gov).

## References

[1] Roth GA Mensah GA Johnson CO Addolorato G Ammirati E Baddour LM. Global burden of cardiovascular diseases and risk factors, 1990-2019: update from the GBD 2019 Study. J Am Coll Cardiol. (2020) 76:2982–3021. 10.1016/j.jacc.2021.02.039 33309175 PMC7755038

[2] Lloyd-Jones DM Hong Y Labarthe D Mozaffarian D Appel LJ Van Horn L. Defining and setting national goals for cardiovascular health promotion and disease reduction: the American heart association's strategic impact goal through 2020 and beyond. Circulation. (2010) 121:586–613. 10.1161/CIRCULATIONAHA.109.192703 20089546

[3] Lloyd-Jones DM Allen NB Anderson CAM Black T Brewer LC Foraker RE. Life's essential 8: updating and enhancing the American heart association's construct of cardiovascular health: a presidential advisory from the American heart association. Circulation. (2022) 146:e18–43. 10.1161/CIR.0000000000001078 35766027 PMC10503546

[4] Chen R Xu J Shang X Bulloch G He M Wang W. Association between cardiovascular health metrics and retinal ageing. Geroscience. (2023) 45:1511–21. 10.1007/s11357-023-00743-3 36930331 PMC10400488

[5] Wu S Wu Z Yu D Chen S Wang A Wang A. Life's essential 8 and risk of stroke: a prospective community-based study. Stroke. (2023) 54:2369–79. 10.1161/STROKEAHA.123.042525 37466001

[6] Chen H Tang H Huang J Luo N Zhang X Wang X. Life's essential 8 and mortality in us adults with chronic kidney disease. Am J Nephrol. (2023) 54:516–27. 10.1159/000533257 37591229

[7] Shen R Zou T. The Association between cardiovascular health and depression: results from the 2007-2020 Nhanes. Psychiatry Res. (2024) 331:115663. 10.1016/j.psychres.2023.115663 38064908

[8] Disease. Global Initiative for Chronic Obstructive Lung. Global Strategy for the Diagnosis, Management, and Prevention of Chronic Obstructive Pulmonary Disease. (2023). Available at: https://Goldcopd.Org/2023-Gold-Report-2/# (accessed November 15, 2022).

[9] Kahnert K Jörres RA Behr J Welte T. The diagnosis and treatment of COPD and its comorbidities. Dtsch Arztebl Int. (2023) 120:434–44. 10.3238/arztebl.m2023.0027 36794439 PMC10478768

[10] Brandsma C-A de Vries M Costa R Woldhuis RR Königshoff M Timens W. Lung ageing and COPD: is there a role for ageing in abnormal tissue repair? Eur Respir Rev. (2017) 26:170073. 10.1183/16000617.0073-2017 29212834 PMC9488745

[11] Postma DS Bush A van den Berge M. Risk factors and early origins of chronic obstructive pulmonary disease. Lancet. (2015) 385:899–909. 10.1016/S0140-6736(14)60446-3 25123778

[12] Rabe KF Hurst JR Suissa S. Cardiovascular disease and COPD: dangerous liaisons? Eur Respir Rev. (2018) 27:180057. 10.1183/16000617.0057-2018 30282634 PMC9488649

[13] Poole DC Richardson RS Haykowsky MJ Hirai DM Musch TI. Exercise limitations in heart failure with reduced and preserved ejection fraction. J Appl Physiol. (2018) 124:208–24. 10.1152/japplphysiol.00747.2017 29051336 PMC5866447

[14] Ponikowski P Voors AA Anker SD Bueno H Cleland JGF Coats AJS. 2016 ESC guidelines for the diagnosis and treatment of acute and chronic heart failure: the task force for the diagnosis and treatment of acute and chronic heart failure of the European Society of Cardiology (ESC) developed with the special contribution of the heart failure association (HFA) of the ESC. Eur Heart J. (2016) 37:2129–200. 10.1093/eurheartj/ehw128 27206819

[15] Patel ARC Donaldson GC Mackay AJ Wedzicha JA Hurst JR. The impact of ischemic heart disease on symptoms, health status, and exacerbations in patients with COPD. Chest. (2012) 141:851–57. 10.1378/chest.11-0853 21940771

[16] Wang L Yi J Guo X Ren X. Associations between life's essential 8 and non-alcoholic fatty liver disease among us adults. J Transl Med. (2022) 20:616. 10.1186/s12967-022-03839-0 36564799 PMC9789599

[17] Lloyd-Jones DM Ning H Labarthe D Brewer L Sharma G Rosamond W. Status of cardiovascular health in us adults and children using the American Heart Association's new “life's essential 8” metrics: prevalence estimates from the national health and nutrition examination survey (NHANES), 2013 through 2018. Circulation. (2022) 146:822–35. 10.1161/CIRCULATIONAHA.122.060911 35766033

[18] Ren Y Cai Z Guo C Zhang Y Xu H Liu L. Associations between Life's Essential 8 and Chronic Kidney Disease. J Am Heart Assoc. (2023) 12:e030564. 10.1161/JAHA.123.030564 38063194 PMC10863789

[19] Zhou R Chen H-W Li F-R Zhong Q Huang Y-N Wu X-B. “Life's essential 8” cardiovascular health and dementia risk, cognition, and neuroimaging markers of brain health. J Am Med Dir Assoc. (2023) 24:1791–97. 10.1016/j.jamda.2023.05.023 37369360

[20] McNicholas WT Hansson D Schiza S Grote L. Sleep in chronic respiratory disease: COPD and hypoventilation disorders. Eur Respir Rev. (2019) 28:190064. 10.1183/16000617.0064-2019 31554703 PMC9488904

[21] Newton K Malik V Lee-Chiong T. Sleep and breathing. Clin Chest Med. (2014) 35:451–6. 10.1016/j.ccm.2014.06.001 25156761

[22] Shen Y Yang T Guo S Li X Chen L Wang T. Increased serum OX-LDL levels correlated with lung function, inflammation, and oxidative stress in COPD. Mediators Inflamm. (2013) 2013:972347. 10.1155/2013/972347 24078777 PMC3774040

[23] Huang Y Ding K Dai Z Wang J Hu B Chen X. The relationship of low-density-lipoprotein to lymphocyte ratio with chronic obstructive pulmonary disease. Int J Chron Obstruct Pulmon Dis. (2022) 17:2175–85. 10.2147/COPD.S369161 36106158 PMC9467295

[24] Li W Ning Y Ma Y Lin X Man S Wang B. Association of lung function and blood glucose level: a 10-year study in China. BMC Pulm Med. (2022) 22:444. 10.1186/s12890-022-02208-3 36434643 PMC9700934

[25] Li J Zhu L Wei Y Lv J Guo Y Bian Z. Association between adiposity measures and COPD risk in Chinese adults. Eur Respir J. (2020) 55:1901899. 10.1183/13993003.01899-2019 31980495 PMC7236866

[26] González P Lozano P Ros G Solano F. Hyperglycemia and oxidative stress: an integral, updated and critical overview of their metabolic interconnections. Int J Mol Sci. (2023) 24:9352. 10.3390/ijms24119352 37298303 PMC10253853

[27] Rains JL Jain SK. Oxidative stress, insulin signaling, and diabetes. Free Radic Biol Med. (2011) 50:567–75. 10.1016/j.freeradbiomed.2010.12.006 21163346 PMC3557825

[28] Cazzola M Rogliani P Ora J Calzetta L Lauro D Matera MG. Hyperglycaemia and chronic obstructive pulmonary disease. Diagnostics (Basel). (2023) 13:3362. 10.3390/diagnostics13213362 37958258 PMC10650064

[29] Barnes PJ. Cellular and molecular mechanisms of chronic obstructive pulmonary disease. Clin Chest Med. (2014) 35:71–86. 10.1016/j.ccm.2013.10.004 24507838

[30] Benz E Trajanoska K Lahousse L Schoufour JD Terzikhan N De Roos E. Sarcopenia in COPD: a systematic review and meta-analysis. Eur Respir Rev. (2019) 28:190049. 10.1183/16000617.0049-2019 31722892 PMC9488535

[31] Agostoni P Cattadori G Guazzi M Palermo P Bussotti M Marenzi G. Cardiomegaly as a possible cause of lung dysfunction in patients with heart failure. Am Heart J. (2000) 140:e24. 10.1067/mhj.2000.110282 11054632

[32] Agostoni P Bussotti M Cattadori G Margutti E Contini M Muratori M. Gas diffusion and alveolar-capillary unit in chronic heart failure. Eur Heart J. (2006) 27:2538–43. 10.1093/eurheartj/ehl302 17028107

[33] Xue X Cai H Chai Z Shang F Guan W Zhang L. Efficacy of statin therapy in chronic obstructive pulmonary disease: a systematic review and meta-analysis from 2008-2019. Panminerva Med. (2023) 65:376–84. 10.23736/S0031-0808.20.03932-4 32343509

[34] Polverino F Wu TD Rojas-Quintero J Wang X Mayo J Tomchaney M. Metformin: experimental and clinical evidence for a potential role in emphysema treatment. Am J Respir Crit Care Med. (2021) 204:651–66. 10.1164/rccm.202012-4510OC 34033525 PMC8521702

[35] Tseng CH. Pioglitazone and risk of chronic obstructive pulmonary disease in patients with type 2 diabetes mellitus: a retrospective cohort study. Int J Chron Obstruct Pulmon Dis. (2022) 17:285–95. 10.2147/COPD.S345796 35177899 PMC8843794

[36] Wang M-T Lai J-H Huang Y-L Kuo F-C Wang Y-H Tsai C-L. Use of antidiabetic medications and risk of chronic obstructive pulmonary disease exacerbation requiring hospitalization: a disease risk score-matched nested case-control study. Respir Res. (2020) 21:319. 10.1186/s12931-020-01547-1 33267895 PMC7709288

[37] Brauwers B Machado FVC Beijers RJHCG Spruit MA Franssen FME. Combined exercise training and nutritional interventions or pharmacological treatments to improve exercise capacity and body composition in chronic obstructive pulmonary disease: a narrative review. Nutrients. (2023) 15:5136. 10.3390/nu15245136 38140395 PMC10747351

