# Supplementary material for: Dose-response relationship between Life's Essential 8 score and COPD risk: the NHANES cohort study 2007–2018
Source: Front Med (Lausanne). 2025 Jan 23;12:1446782. doi: 10.3389/fmed.2025.1446782 (PMC11801010; doi:10.3389/fmed.2025.1446782)
Supplement: Supplementary file 1 [file Table_1.docx]

| **V**ariable | **Pre-PSM** | | ***P*** | **Post-PSM** | | ***P*** |
| --- | --- | --- | --- | --- | --- | --- |
|  | **Low(** 0-49) | **Moderate**( 50-79) |  | **Low(** 0-49) | **Moderate**( 50-79) |  |
| **S**ex(%) |  |  | 0.05 |  |  | 0.92 |
| Female | 1117(54.74) | 4300(50.91) |  | 1117(54.74) | 1122(56.80) |  |
| Male | 981(45.26) | 4329(49.09) |  | 981(45.26) | 976(43.20) |  |
| **Age group(%)** |  |  | 0.42 |  |  | 0.79 |
| **40-64** | 1361(73.22) | 5940(74.38) |  | 1361(73.22) | 1373(71.15) |  |
| **≥65** | 737(26.78) | 2689(25.62) |  | 737(26.78) | 725(28.85) |  |
| **Race**(%) |  |  | < 0.001 |  |  | 0.89 |
| **O**ther | 1218(33.43) | 4843(28.02) |  | 1218(33.43) | 1210(29.82) |  |
| White | 880(66.57) | 3786(71.98) |  | 880(66.57) | 888(70.18) |  |
| **M**arital status (%) |  |  | <0.0001 |  |  | 0.35 |
| Married/Living with Partner | 1127(58.37) | 5454(67.62) |  | 1127(58.37) | 1197(61.35) |  |
| Never married | 215(10.42) | 721( 7.59) |  | 215(10.42) | 191( 8.32) |  |
| Widowed/Divorced/Separated | 756(31.20) | 2454(24.79) |  | 756(31.20) | 710(30.32) |  |
| **Education(%)** |  |  | <0.0001 |  |  | 0.99 |
| High school diploma | 580(32.47) | 2109(26.44) |  | 580(32.47) | 583(34.40) |  |
| Lower than high school | 725(25.84) | 2084(14.99) |  | 725(25.84) | 727(23.68) |  |
| More than high school | 793(41.68) | 4436(58.57) |  | 793(41.68) | 788(41.92) |  |
| **Poverty income ratio(%)** |  |  | <0.0001 |  |  | 1 |
| <1.3 | 886(34.14) | 2415(17.13) |  | 886(34.14) | 886(28.97) |  |
| 1.3-3.5 | 858(39.40) | 3338(35.51) |  | 858(39.40) | 860(42.03) |  |
| **>3.5** | 354(26.46) | 2876(47.35) |  | 354(26.46) | 352(29.00) |  |
| **Insurance**(%) |  |  | 0.002 |  |  | 0.75 |
| No | 360(16.64) | 1445(12.66) |  | 360(16.64) | 372(15.37) |  |
| Yes | 1738(83.36) | 7184(87.34) |  | 1738(83.36) | 1726(84.63) |  |

**Table 4. Low and moderate level LE8 score propensity score matches and results.**

**Table 5. Moderate and high level LE8 score propensity score matches and results.**

| **Variable** | **Pre-PSM** | | ***P*** | **Post-PSM** | | ***P*** |
| --- | --- | --- | --- | --- | --- | --- |
|  | **Moderate(50-79)** | **High (80-100)** |  | **Moderate(50-79)** | **High (80-100)** |  |
| **Sex(%)** |  |  | <0.0001 |  |  | 0.25 |
| **Female** | 4300(50.91) | 1088(60.15) |  | 2083(55.73) | 1088(60.15) |  |
| **Male** | 4329(49.09) | 702(39.85) |  | 1497(44.27) | 702(39.85) |  |
| **Age group(%)** |  |  | <0.0001 |  |  | 0.93 |
| **40-64** | 5940(74.38) | 1425(83.11) |  | 2845(81.57) | 1425(83.11) |  |
| **≥65** | 2689(25.62) | 365(16.89) |  | 735(18.43) | 365(16.89) |  |
| **Race(%)** |  |  | <0.0001 |  |  | 0.86 |
| **Other** | 4843(28.02) | 893(21.05) |  | 1797(21.95) | 893(21.05) |  |
| **White** | 3786(71.98) | 897(78.95) |  | 1783(78.05) | 897(78.95) |  |
| **Marital status (%)** |  |  | <0.0001 |  |  | 0.003 |
| **Married/Living with Partner** | 5454(67.62) | 1315(78.16) |  | 2406(71.52) | 1315(78.16) |  |
| **Never married** | 721(7.59) | 133(5.78) |  | 301(7.02) | 133(5.78) |  |
| **Widowed/Divorced/Separated** | 2454(24.79) | 342(16.06) |  | 873(21.47) | 342(16.06) |  |
| **Education(%)** |  |  | <0.0001 |  |  | 0.97 |
| **High school diploma** | 2109(26.44) | 242(11.48) |  | 493(14.32) | 242(11.48) |  |
| **Lower than high school** | 2084(14.99) | 210( 5.99) |  | 413(5.64) | 210(5.99) |  |
| **More than high school** | 4436(58.57) | 1338(82.53) |  | 2674(80.05) | 1338(82.53) |  |
| **Poverty income ratio(%)** |  |  | <0.0001 |  |  | 0.58 |
| **<1.3** | 2415(17.13) | 279( 8.80) |  | 551(8.72) | 279(8.80) |  |
| **1.3-3.5** | 3338(35.51) | 550(24.65) |  | 1172(27.03) | 550(24.65) |  |
| **>3.5** | 2876(47.35) | 961(66.54) |  | 1857(64.25) | 961(66.54) |  |
| **Insurance(%)** |  |  | 0.004 |  |  | 0.83 |
| **No** | 1445(12.66) | 223( 9.51) |  | 455(8.05) | 223(9.51) |  |
| **Yes** | 7184(87.34) | 1567(90.49) |  | 3125(91.95) | 1567(90.49) |  |

**Table 6. Relationship between LE8 score and COPD after propensity score matching.**

| **LE8 score** | **OR (95%CI)** | ***P*** |
| --- | --- | --- |
| **Low (0-49)** | Reference |  |
| **Moderate (50-79)** | 0.39(0.29,0.53) | <0.0001 |
| **Moderate (50-79)** | Reference |  |
| **High (80-100)** | 0.42(0.26,0.67) | <0.001 |

**Table 7. Sensitivity analysis baseline characteristics**

| **Variable** | **Total** | **Non-COPD** | **COPD** | ***P*** |
| --- | --- | --- | --- | --- |
| **Sex(%)** |  |  |  | 0.03 |
| **Female** | 5391(53.23) | 5074(53.78) | 317(45.58) |  |
| **Male** | 5046(46.77) | 4623(46.22) | 423(54.42) |  |
| **Age group(%)** |  |  |  | < 0.0001 |
| **40-64** | 7251(75.88) | 6824(76.59) | 427(66.17) |  |
| **≥65** | 3186(24.12) | 2873(23.41) | 313(33.83) |  |
| **Race(%)** |  |  |  | < 0.0001 |
| **Other** | 5718(27.29) | 5444(27.96) | 274(18.02) |  |
| **White** | 4719(72.71) | 4253(72.04) | 466(81.98) |  |
| **Marital status (%)** |  |  |  | 0.12 |
| **Married/Living with Partner** | 6551(67.74) | 6127(67.85) | 424(66.33) |  |
| **Never married** | 898( 7.81) | 844(7.95) | 54(5.95) |  |
| **Widowed/Divorced/Separated** | 2988(24.45) | 2726(24.21) | 262(27.71) |  |
| **Education(%)** |  |  |  | < 0.0001 |
| **High school diploma** | 2455(24.94) | 2258(24.72) | 197(27.94) |  |
| **Lower than high school** | 2547(14.98) | 2327(14.57) | 220(20.74) |  |
| **More than high school** | 5435(60.08) | 5112(60.71) | 323(51.32) |  |
| **Poverty income ratio(%)** |  |  |  | < 0.0001 |
| **<1.3** | 2961(17.74) | 2691(17.22) | 270(25.01) |  |
| **1.3-3.5** | 3969(34.21) | 3677(33.78) | 292(40.15) |  |
| **>3.5** | 3507(48.05) | 3329(49.00) | 178(34.84) |  |
| **Insurance(%)** |  |  |  | 0.26 |
| **No** | 1722(13.05) | 1639(13.19) | 83(11.03) |  |
| **Yes** | 8715(86.95) | 8058(86.81) | 657(88.97) |  |
| **Life's Essential 8(%)** |  |  |  | < 0.0001 |
| **0-49** | 1753(13.50) | 1526(12.70) | 227(24.56) |  |
| **50-79** | 7217(68.38) | 6737(68.28) | 480(69.73) |  |
| **80-100** | 1467(18.12) | 1434(19.01) | 33( 5.70) |  |
| **Blood lead(%)** |  |  |  | < 0.0001 |
| **[0.05, 0.9]** | 2641(28.78) | 2533(29.71) | 108(15.97) |  |
| **(0.9, 1.34]** | 2620(26.64) | 2460(26.96) | 160(22.24) |  |
| **(1.34, 2.04]** | 2586(23.74) | 2402(23.62) | 184(25.38) |  |
| **(2.04, 38.9]** | 2590(20.84) | 2302(19.71) | 288(36.41) |  |
| **Blood mercury(%)** |  |  |  | 0.31 |
| **[0.11, 0.47]** | 2642(24.31) | 2425(24.03) | 217(28.16) |  |
| **(0.47, 0.88]** | 2588(23.92) | 2378(23.86) | 210(24.78) |  |
| **(0.88, 1.77]** | 2599(25.81) | 2450(26.09) | 149(21.88) |  |
| **(1.77, 85.7]** | 2608(25.96) | 2444(26.01) | 164(25.18) |  |
| **Asthma** |  |  |  | < 0.0001 |
| **No** | 9061(86.85) | 8642(88.97) | 419(57.56) |  |
| **Yes** | 1376(13.15) | 1055(11.03) | 321(42.44) |  |

**Table 8. Sensitivity analysis of the association between the LE8 score and COPD.**

|  | **Model1** | ***P*** | **Model2** | ***P*** | **Model3** | ***P*** |
| --- | --- | --- | --- | --- | --- | --- |
|  | **OR (95%CI)** |  | **OR (95%CI)** |  | **OR (95%CI)** |  |
| **LE8 score** |  |  |  |  |  |  |
| **Low (0-49)** | Reference | / | Reference | / | Reference | / |
| **Moderate (50-79)** | 0.53(0.42,0.66) | <0.0001 | 0.53(0.42,0.67) | <0.0001 | 0.66(0.52,0.85) | 0.002 |
| **High (80-100)** | 0.16(0.09,0.26) | <0.0001 | 0.17(0.10,0.29) | <0.0001 | 0.22(0.12,0.40) | <0.0001 |
| ***P for trend*** | <0.0001 | / | <0.0001 | / | <0.0001 | / |
